# Accuracy of CT angiography for detecting ruptured intracranial aneurysms

**DOI:** 10.4102/sajr.v27i1.2636

**Published:** 2023-05-30

**Authors:** Nomasonto N. Mkhize, Victor Mngomezulu, Thandi E. Buthelezi

**Affiliations:** 1 Department of Diagnostic Radiology, Faculty of Health Sciences, University of the Witwatersrand, Johannesburg, South Africa; 2 Department of Diagnostic Radiology, Chris Hani Baragwanath Academic Hospital, Johannesburg, South Africa

**Keywords:** digital subtraction angiography, DSA, computed tomography angiography, CTA, intracranial aneurysms, ruptured aneurysms, comparison, accuracy, diagnosis

## Abstract

**Background:**

Digital subtraction angiography (DSA) is invasive, costly and unavailable in many South African hospitals; however, it remains the gold standard for imaging intracranial aneurysms. CT angiography (CTA) is a non-invasive and readily available screening tool prior to DSA.

**Objectives:**

This study aimed to evaluate the diagnostic performance of CTA in detecting ruptured intracranial aneurysms using DSA as the reference standard and to determine the effect of aneurysm size and location on CTA sensitivity.

**Method:**

A retrospective analysis of CTA and DSA data from reports of patients suspected to have aneurysmal subarachnoid haemorrhage (SAH) at Chris Hani Baragwanath Academic Hospital between January 2017 and June 2020.

**Results:**

Conventional DSA detected aneurysms in 94 out of 115 patients; while of these, CTA detected 75 and missed 19. The CTA sensitivity, specificity and accuracy was 80%, 43% and 73%, respectively. The CTA sensitivity for aneurysms < 3 mm and 3 mm – 5 mm in size was 30% and 81.5%, respectively (*p* = 0.024). Sensitivity of CTA for posterior communicating artery (PComm) aneurysms was 56% and lower than other major anterior circulation locations (83% – 91%) (*p* = 0.045).

**Conclusion:**

The CTA diagnostic efficiency was lower than previously reported, with even lower sensitivity for aneurysms < 3 mm and for those arising from the PComm. Thus, CTA should remain a screening tool prior to DSA in all local patients suspected to have aneurysmal SAH.

**Contribution:**

Larger, prospective studies are required to accurately define the role of CTA in diagnosing intracranial aneurysms in a developing country with limited resources.

## Introduction

Subarachnoid haemorrhage (SAH) resulting from a ruptured intracranial aneurysm is a devastating haemorrhagic stroke accounting for 5% – 15% of all strokes.^1,2^ This type of stroke has a high mortality rate and significant morbidity in survivors.^2,3,4,5^ Rapid diagnosis and triage of patients to appropriate management is therefore crucial to improve clinical outcome.

Conventional, intra-arterial catheter digital subtraction angiography (DSA) is the gold standard for diagnosing and characterising intracranial aneurysms because of its excellent spatial and temporal resolution.^2,4^ Diagnostic DSA is, however, an invasive, costly investigation, which requires specialised neuro-interventional expertise and equipment.^2,4^ Furthermore, DSA carries a low (less than 1%) but real risk of serious neurologic, systemic and local complications.^6,7^

Cerebral CT angiography (CTA) is non-invasive, affordable and can be easily performed after a non-contrast CT brain demonstrating non-traumatic SAH. In our institution, Chris Hani Baragwanath Academic Hospital (CHBAH), CTA is used as a screening vascular imaging modality prior to DSA. The latter is subsequently performed at the earliest possible opportunity depending on resource availability and the patient’s condition.

As a result of advances in multidetector computed tomography (MDCT) technology, there has been interest in defining the role of CTA in the diagnosis and characterisation of intracranial aneurysms, as a potential alternative to DSA in certain cases.^8,9^ The majority of data in the literature showed that CTA has a good diagnostic efficiency which is comparable to DSA with a high sensitivity, specificity and accuracy of 95% – 98%, 90% – 100% and 94% – 98%, respectively.^10,11,12,13^ Meta-analyses of up to 50 studies stated similar findings with pooled sensitivities and specificities of 97% – 98% and 98% – 100%, respectively.^8,9^ A few studies demonstrated lower CTA diagnostic performance with overall sensitivity of 71% – 89% and accuracy of 74% – 90%.^14,15^

The reduction in CTA detection efficiency with decrease in aneurysm size is well documented in the literature.^11,15,16,17^ A threshold of 3 mm has been described by several authors as a pitfall for aneurysm detection on CTA, with sensitivity as low as 35% – 45% for aneurysms ≤ 3 mm, in contrast to sensitivity of 81% – 99% for aneurysms > 3 mm in size.^15,16^ Other authors reported better sensitivity (74% – 87%) for very small aneurysms (< 3 mm) but the pattern of improved sensitivity with increase in size was still evident (92% – 98%) for aneurysms ≥ 3mm.^11,13,18^ Aneurysms in certain locations such as the posterior communicating artery (PComm)^19^ and internal carotid artery (ICA) were difficult to detect on CTA because of their proximity to the base of skull bony structures and aneurysms from smaller calibre arteries.^14,15,17^

A single, published South African study describing CTA and DSA findings in various causes of intracranial haemorrhage, including trauma, aneurysms, vasculitis and vascular malformations, found that CTA had a diagnostic yield comparable to DSA for aneurysms, but DSA was superior for multiple aneurysms.^20^ These results were supported by several previous studies.^15,17^

There is a need to define the role of CTA in our environment, as a potential alternative to diagnostic DSA in specific cases. The aims and objectives of this study were to evaluate the diagnostic efficiency of CTA in detecting ruptured intracranial aneurysms using DSA as a reference standard by calculating its sensitivity, specificity, positive predictive value (PPV), negative predictive value (NPV) and accuracy and also, to determine the effect of aneurysm size and location on CTA detection efficiency.

## Research methods and design

A retrospective, cross-sectional audit of CTA and DSA reports of patients suspected of aneurysmal SAH was performed. Reports of 403 patients between January 2017 and June 2020 were retrieved from the Chris Hani Baragwanath Academic Hospital picture archiving communication system (PACS) and analysed.

The Fleiss formula was used to calculate a representative sample size of 114 patients at a 95% confidence interval (CI) and 80% power. A total of 115 adult patients (≥ 18 years) who underwent both CTA and DSA were included in the study. All CTA reports were reported or approved by a specialist radiologist and all DSA reports were reported by a neuro-interventional radiologist. Cases with inadequate data on either of the reports or previously treated aneurysms were excluded. Patients found to have other identifiable causes for SAH such as arteriovenous malformation (AVM) or vasculitis, were also excluded.

The cerebral CTA images were acquired on one of the three MDCT scanners: Philips Ingenuity CT 128, Toshiba Aquilion CT 128 or Toshiba Aquilion CT 64. The 4-vessel cerebral DSA images were acquired on the Philips Allura X-Per Biplane C-arm fluoroscopy machine. All patients had the CTA prior to the DSA with a mean interval time of 6.5 days (± 3.1).

Data were extracted from CTA and DSA reports and populated onto an Excel spreadsheet. The data included patients’ demographics (age and gender), presence or absence of causative aneurysm on both modalities, additional aneurysm(s), and size and location of aneurysms. The size of aneurysm was the maximum diameter measured on DSA. Aneurysm diameter was categorised into size groups, adapted from Philipp et al. and Meyers et al. as follows: very small (< 3 mm), small (≥ 3 mm to ≤ 5mm), medium-sized (> 5 mm to < 15 mm), large (≥ 15 mm to < 25 mm) and giant (≥ 25 mm).^14,21^ The aneurysm location was classified broadly into anterior and posterior circulation and further categorised per vessel of origin as demonstrated by DSA.

### Statistical analysis

The continuous variables were presented as means ± standard deviation or median with interquartile range (IQR) where appropriate. Categorical variables were expressed as frequencies and percentages. The CTA sensitivity, specificity, NPV, PPV and accuracy was calculated on per patient and per aneurysm-bases using two-by-two contingency tables, with DSA as the reference standard. An appropriate CI of 95% was used. Furthermore, CTA sensitivity, aneurysm frequency and proportions were calculated for various aneurysm size groups and locations. Comparison of sensitivity for various aneurysm size groups and locations was performed using the Pearson’s Chi-squared test and Fisher’s exact test, adjusted using the Bonferroni correction. The statistically significant difference was set at a *p*-value < 0.05 with a 95% CI. Statistical analysis was performed on STATA^®^ version 15 (Stata Corp).

## Results

A total of 115 patients were included in this study, aged between 19 and 83 years (mean: 46 years ± 13.3), with the majority in the 41–50 and 51–60-year age groups (Table 1). The female to male ratio was 1.5:1 (Table 1). Digital subtraction angiography detected ruptured aneurysms in 94 of 115 patients. Fourteen of the 94 patients (15%) had multiple aneurysms. Of the 14 patients, 10 patients had one additional aneurysm, three patients had two aneurysms and one patient had three additional aneurysms. In total, DSA detected 113 ruptured and unruptured aneurysms in 94 patients. Therefore, 94 aneurysms per patient-basis and 113 aneurysms per aneurysm-basis were detected by the gold standard (DSA) (Table 2). No identifiable cause of SAH was found in 21 patients (18%).

**TABLE 1:** Summary of sample patients demographics.

Variables	Number of patients	
	*n*	%
**Gender**		
Female	69	60
Male	46	40
**Age (years)**		
< 20	1	0.9
20–30	16	13.9
31–40	22	19.1
41–50	34	29.6
51–60	26	22.6
61–70	13	11.3
> 70	3	2.6

**TABLE 2:** Overall diagnostic performance of CT angiography using digital subtraction angiography as the reference standard per patient-basis and per aneurysm-basis.

Aneurysms	CTA findings relative to DSA (gold standard)				Statistical analysis				
	TP	FN	TN	FP	Sensitivity (%)	Specificity (%)	PPV (%)	NPV (%)	Accuracy (%)
Per patient-basis DSA positive (*n* = 94)	75	19	9	12	79.8	42.9	86.2	32.1	73.0
Per aneurysm-basis DSA positive (*n* = 113)	75	38	9	12	66.4	42.9	86.2	19.0	62.7

*Source*: Adapted from Lu L, Zhang LJ, Poon CS, et al. Digital subtraction CT angiography for detection of intracranial aneurysms: Comparison with three-dimensional digital subtraction angiography. Radiology. 2012;262(2):605–612. https://doi.org/10.1148/radiol.11110486

PPV, positive predictive value; NPV, negative predictive value; TP, true positive; FN, false negative; TN, true negative; FP, false positive; CTA, computed tomography angiography.

### Overall CT angiography diagnostic performance

CT angiography correctly identified 75 of 94 (80%) and missed 19 of 94 (20%) ruptured aneurysms per patient-bases. The detailed diagnostic performance of CTA per patient-basis is demonstrated in Table 2. Computed tomography angiography missed all 19 incidental, non-causative aneurysms in 14 patients with multiple aneurysms on DSA, resulting in a higher false negative (FN) finding of 38 aneurysms. Thus, the diagnostic performance of CTA per aneurysm-basis was poor, with a decreased sensitivity and accuracy of 66% and 63%, respectively.

### Aneurysm size

**FIGURE 1:**
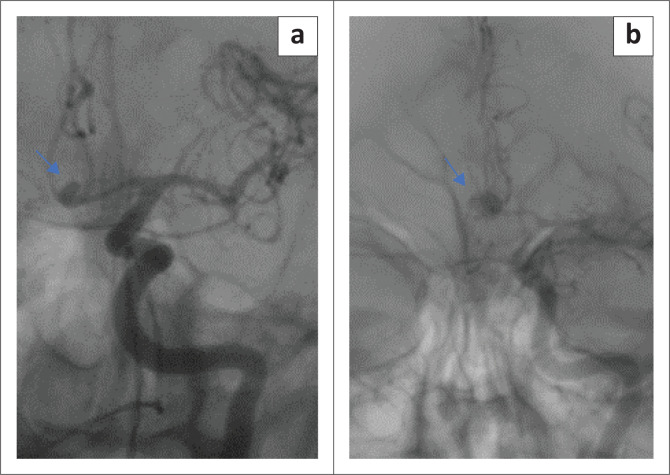
Left internal carotid artery angiogram (non-subtracted images) frontal views of different patients. (a) Very small (2.8 mm) saccular aneurysm arising from the anterior communicating artery (arrow), which was missed on computed tomography angiography, in a 62-year-old male. (b) Small (4.4 mm) saccular aneurysm also arising from the anterior communicating artery (arrow), which was correctly identified on computed tomography angiography, in a 54-year-old female.

Statistical analysis regarding aneurysm size and location was performed on per patient-basis, which was 94 aneurysms considered to be ruptured in 94 patients. The aneurysm size ranged from 1 mm to 30 mm, with a median of 5.8 mm (IQR: 4.0–8.3). The frequency distribution of aneurysms by size group as determined by DSA and the respective CTA detection efficiency are demonstrated in Table 3. The large and giant aneurysms were all correctly identified on CTA; however, they were very few in number (6/94), therefore, were excluded from comparative statistical analysis. Computed tomography angiography missed 7 of 10 (70%) very small aneurysms (< 3 mm) resulting in a poor sensitivity of 30%. The CTA sensitivity (30%) for very small aneurysms (< 3 mm) was significantly poor compared with the 3–5 mm (small size) group, the > 5 mm to < 15 mm (medium-sized) group and the overall CTA sensitivity (80%) (*p* = 0.024). Figure 1 shows a very small and a small aneurysm.

**TABLE 3:** CT angiography detection efficiency by aneurysm size category.

Aneurysm size (mm)	DSA positive (gold standard) (*n* = 94)		Per patient bases		
	*n* (*n* = 94)	%	CTA Positive (true positive) (*n* = 75)	CTA Negative (false negative) (*n* = 19)	CTA sensitivity (%)
Very small (< 3)	10	10.6	3	7	30*
Small (≥ 3 to ≤ 5)	27	28.7	22	5	81.5
Medium (> 5 to < 15)	51	54.2	44	7	86.3
Large (≥ 15 to < 25)	*2*	*2.1*	*2*	*0*	*100.0*
Giant (≥ 25)	*4*	*4.2*	*4*	*0*	*100.0*

*Source:* Adapted from Philipp LR, McCracken DJ, McCracken CE, et al. Comparison between CTA and digital subtraction angiography in the diagnosis of ruptured aneurysms. Neurosurgery. 2017;80(5):769–777. https://doi.org/10.1093/neuros/nyw113

Note: Data in italics indicate size groups with too few aneurysms for accurate comparative analysis.

DSA, digital subtraction angiography; CTA, computed tomography angiography.

* , *p*-value = 0.024.

### Aneurysm location

The frequency distribution of aneurysms by vessel of origin and their respective detectability on CTA is demonstrated in Table 4. The pericallosal artery, callosomarginal artery, anterior choroidal artery, posterior inferior cerebellar artery (PICA), basilar tip, anterior inferior cerebellar artery (AICA) and vertebral artery had the minority of aneurysms, each location with three or less aneurysms, thus these locations were not included in the comparative statistical analysis.

**TABLE 4:** CT angiography detection efficiency by aneurysm location.

Aneurysm location	Per patient bases				
	DSA positive (gold standard)		CTA positive (true positive) (*n*)	CTA negative (false negative)	CTA (Sensitivity)

	*n*	%	*n*	*n*	%
**Anterior circulation**	87	93.0	69	18	79.3
AComm	22	23.4	20	2	90.9
ICA	18	19.1	15	3	83.3
PComm	16	17.0	9	7	56.2*
MCA	14	14.9	12	2	85.7
ACA	10	10.6	9	1	90.0
Pericallosal A	*3*	*3.2*	*2*	*1*	*66.7*
Ant Choroidal A	*3*	*3.2*	*1*	*2*	*33.3*
Callosomarginal A	*1*	*1.1*	*0*	*1*	*0.0*
**Posterior circulation**	7	7.0	6	1	85.7
PICA	*3*	*3.2*	*2*	*1*	*66.7*
Basilar tip	*2*	*2.1*	*2*	*0*	*100.0*
AICA	*1*	*1.1*	*1*	*0*	*100.0*
Vertebral artery	*1*	*1.1*	*1*	*0*	*100.0*

**Total aneurysms (*N*)**	**94**	-	**75**	**19**	-

*Source:* Adapted from Philipp LR, McCracken DJ, McCracken CE, et al. Comparison between CTA and digital subtraction angiography in the diagnosis of ruptured aneurysms. Neurosurgery. 2017;80(5):769–777. https://doi.org/10.1093/neuros/nyw113

Note: Data in italics are locations with too few aneurysms for accurate comparative analysis.

DSA, digital subtraction angiography; AComm, anterior communicating artery; ICA, internal carotid artery; PComm, posterior communicating artery; MCA, middle cerebral artery; ACA, anterior cerebral artery; PICA, posterior inferior cerebellar artery; AICA, anterior inferior cerebellar artery.

* , *p*-value = 0.045.

There was no statistically significant difference in CTA sensitivity for aneurysms arising from the anterior versus the posterior circulation (*p* = 0.71), however, the posterior circulation aneurysms were remarkably fewer in number. The CTA sensitivity (56.2%) for aneurysms arising from PComm was significantly poor compared with the rest of the major anterior circulation locations (AComm, ACA, MCA and ICA) (83% – 91%) and the overall CTA sensitivity (80%) (*p* = 0.045).

## Discussion

In this single, tertiary institution study we retrospectively evaluated the diagnostic accuracy of CTA for detecting ruptured intracranial aneurysms using DSA as a reference standard. The CTA diagnostic performance was limited, with sensitivity, specificity, PPV, NPV and accuracy of 80%, 43%, 86%, 32% and 73%, respectively. The CTA diagnostic efficiency was even lower for multiple aneurysms because of failure to detect all additional, non-causative aneurysms. Very small aneurysms with diameters < 3 mm were difficult to detect on CTA (sensitivity of 30%), while there was a drastic improvement in sensitivity for aneurysms ≥ 3 mm with sensitivities from 81.5%. There was no significant difference between the CTA sensitivity for anterior versus posterior circulation aneurysms, even though the vast majority of aneurysms were located in the anterior circulation. Aneurysms arising from PComm were difficult to detect on CTA (sensitivity of 56%), whereas CTA sensitivity was not adversely affected in the rest of the major anterior circulation locations, namely anterior communicating artery (AComm), anterior cerebral artery (ACA), middle cerebral artery (MCA) and internal carotid artery (ICA).

The vast majority of previous studies in the literature showed good CTA diagnostic performance that is comparable to DSA.^8,9,10,11,12,13,17^ This is contrary to our study, which showed lower CTA performance than previous reports. The compromised diagnostic performance of CTA in our study was related to unexpectedly high false negative and false positive CTA results in 19 and 12 patients, respectively. Factors that may have contributed to these false findings include the heterogeneity of CTA reporting by various radiologists with a wide range of experience in contrast to DSA images, which were reported by an experienced neuro-radiologist; and inadequacy of CTA bone subtraction and volume rendering software on PACS versus optimal digital subtraction in the angio-suite viewing station. In addition, false positive findings may be attributed to vessel tortuosity, adjacent venous filling or anatomical variants mimicking aneurysmal outpouchings. These factors were also considered and acknowledged by other authors.^15,16,17^

Good CTA performance reported in the literature may be because of optimal reading conditions by at least two experienced neuro-radiologists reading CTA and DSA images either individually or in consensus^11,12,13,17^ and consistent use of post processing tools and techniques. Some studies used a probability scale for the level of confidence in diagnosing the aneurysms on CTA.^10,17^ Contrasting this, few previous studies showed limited overall CTA diagnostic performance^14,15^ similar to this study. Compromise in CTA sensitivity was demonstrated for multiple, incidental aneurysms in this study, congruent to several studies.^15,17^ The low NPV (32%) in this study depicts CTA as a suboptimal screening test, similar to Philip et al. (50%)^14^ but contrary to most studies that demonstrated NPVs of 91% and higher.^11,16,17^

**FIGURE 2:**
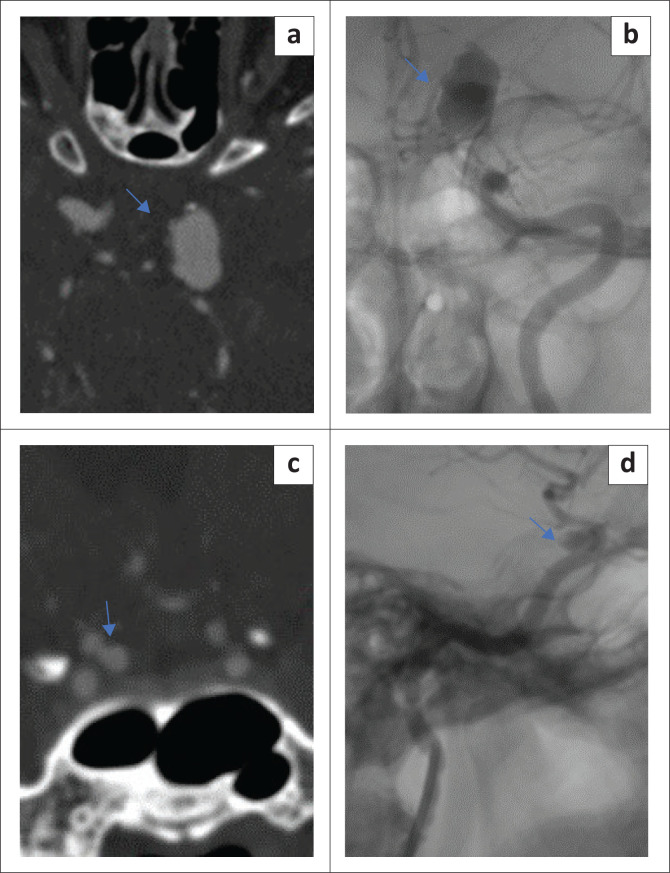
(a) CT angiography axial and (b) left internal carotid artery angiogram (non-subtracted image) frontal view showing a medium-sized (13 mm) fusiform aneurysm arising from the supraclinoid segment of left internal carotid artery (arrows) in a 65-year-old female. (c) CT angiography coronal maximum intensity projection (MIP) and (d) right internal carotid artery angiogram (non-subtracted image) lateral view showing a small (4.6 mm) saccular aneurysm arising from the medial aspect of supraclinoid segment of right internal carotid artery.

Diminished CTA sensitivity for very small aneurysms (≤ 3 mm) is well documented in the literature,^11,15,17^ which is congruent to this study. In contrast Lu et al. demonstrated good CTA sensitivity even for aneurysms < 3 mm in size using dual energy CTA.^12^ Some authors reported no difference in CTA sensitivity for anterior versus posterior circulation aneurysms.^11,12^ This finding is supported by this study, even though the aneurysm frequency proportions were markedly skewed towards the anterior circulation. Several studies reported lower CTA sensitivity for locations close to the bony base of skull such as the ICA^14,15^ and PComm.^19^ The latter was congruent with the findings of this study, which demonstrated significantly lower PComm sensitivity (56%) compared with other major anterior circulation locations. The ICA location however did not show significantly compromised CTA sensitivity in this study (Figure 2).

### Limitations

Limitations in this study may have contributed to the lower CTA diagnostic efficiency compared with the literature data, despite the use of 64 and 128 row MDCT scanners similar to most previous studies. These limitations include the retrospective analysis of reports that were conducted as part of patient investigation; no repeat image reading was performed. The image quality of the CTA studies was not evaluated for degradation because of movement artifact or inadequate contrast opacification of the arteries or venous filling. Radiologists with different experience levels reported or approved the CTA images whereas DSA was reported by an experienced interventional radiologist. The use of CT post processing techniques (MIP, 3D reconstruction, bone subtraction techniques) during image interpretation of the CTA were not routinely documented. There were a limited number of patients particularly for comparisons in the size and location subcategories.

## Conclusion

In this study CTA was less accurate in detecting intracranial aneurysms than previously reported. Its detection efficiency was further compromised for very small aneurysms of < 3 mm in diameter, aneurysms located in the posterior communicating artery and for multiple aneurysms. The overall CTA diagnostic accuracy for intracranial aneurysms is inferior to the gold standard (diagnostic DSA). Therefore, CTA should remain a first-line, adjunct, vascular imaging modality prior to DSA in all cases of suspected aneurysmal SAH locally. There is, however, a need for larger cohort and prospective studies to accurately define the role of CTA in this clinical setting in a developing country with limited resources, paying particular attention to factors such as double reading of CTA images and consistent use of post processing tools.
